# Effects of aGVHD and cGVHD on Survival Rate in Patients with Acute Myeloid Leukemia after Allogeneic Stem Cell Transplantation

**Published:** 2015-07-01

**Authors:** Shabnam Shokouhi, Sarah Bray, Salar Bakhtiyari, Kourosh Sayehmiri, Kamran Alimoghadam, Ardeshir Ghavamzadeh

**Affiliations:** 1 Department of Epidemiology, Faculty of Public Health, Ilam University of Medical Sciences, Ilam, Iran; 2 Psychosocial Injuries Research Center, Ilam University of Medical Sciences, Ilam, Iran; 3 Discipline of Medicine, Faculty of Health Sciences, University of Adelaide, Adelaide, South Australia, Australia; 4 Department of Hematology/Oncology, Basil Hetzel Institute for Translational Health Research, The Queen Elizabeth Hospital, Woodville, South Australia, Australia; 5 Department of Clinical Biochemistry, Faculty of Medicine, Ilam University of Medical Sciences, Ilam, Iran; 6 Hematology-Oncology and Stem Cell Transplantation Research Center, Tehran University of Medical Sciences, Tehran, Iran

**Keywords:** Acute Myeloid Leukemia, cGVHD, aGVHD, HSCT, Survival rate

## Abstract

**Background:** Allogeneic Hematopoietic Stem Cell Transplantation (HSCT) is a curative treatment option for many patients with Acute Myeloid Leukemia (AML); however, it can lead to complications of Graft-Versus-Host-Disease (GVHD) which can affect the quality of life and overall survival. The aim of this study was to assess the effects of both acute and chronic GVHD on survival rate in patients with AML who received HSCT.

**Subjects and Methods:** In a longitudinal study, 587 patients with AML who underwent bone marrow transplantation in Tehran-Iran between1991 and 2011 were recruited. All patient records were analyzed for the occurrence of adverse events including acute and chronic GVHD and leukemia relapse. Data were analyzed using Log-rank, Kaplan-Meier, Univariate and Multivariate Cox Regression models.

**Results:** The five-year overall survival (OS) was found to be 71.9% (95% CI: 67.40-76.41). Also there was a significant relationship between cGVHD and OS (P=0.001, HR = 0.476, 95%). Hazard of death in these patients was less than those who did not experience an occurrence of cGVHD and aGVHD (HR= 0.629, P= 0.078). A significant relationship between cGVHD and relapse was observed (P< 0.001) indicating that patients who developed cGVHD experienced a better survival rate. A significant relationship was also found between overall survival and aGVHD grade (P< 0.001). Hazard of death (HD) for cGVHD and relapse variables were estimated to be 0.554 and 3.869.

**Discussion:** This study is one of the largest studies (regarding the number of participants) done to date in the Middle East with quite a long duration (20 years). cGVHD appears to have a positive influence on survival rate in patients with AML who received HSCT. It is recommended that further studies investigate the underlying reason or mechanisms behind this.

## Introduction

 Allogeneic Hematopoietic Stem Cell Transplantation (HSCT) is a curative treatment option for many patients with acute myeloid leukemia (AML). The effectiveness of HSCT treatment arises through a combination of the cytotoxic effects of the pre-transplantation conditioning regimen and the immunological effect of transplanted donor cells reacting against the host malignant cells, termed the ‘graft-versus-leukemia’ (GVL) effect. 

Despite the success of HSCT, transplantation-related complications such as graft-versus-host-disease (GVHD) frequently occur, and can adversely affect the quality of life and overall survival.^1^^-^^3^ GVHD occurs when the donor immune cells from the HSCT recognize the normal host tissue as foreign, and mount an immunological attack against the host. GVHD historically can be classified into two forms: acute (aGVHD) and chronic (cGVHD), based on whether the disease arises before or after the 100^th^ day post transplantation.^4^

The occurrence of GVHD, and lack of predictive tests to determine which patients are at greater risk of developing severe GVHD are still factors preventing more widespread use of HSCT, particularly in older and frail AML patients. However, the situation is further complicated with some studies reporting that AML patients experiencing GVHD following HSCT actually have a lower risk of relapse compared to patients who did not experience GVHD, while other studies have failed to find any such beneficial relationship between GVHD and relapse.^5^^-^^9^

In this study, the incidence and effects on survival rates of both acute and chronic GVHD on 587 AML patients (aged <60 years) who underwent HSCT in Iran during a twenty-year period between 1991 and 2011 were evaluated. To the best of the researchers’ knowledge this cohort represents the largest transplantation dataset in the Middle East, and thus provides a good opportunity to investigate the occurrence and severity of GVHD and impact on outcomes in a large number of patients. 

## SUBJECTS AND METHODS

**Patients**

 Samples used in this study were obtained from a larger longitudinal study on 1012 patients with acute lymphoblastic leukemia (ALL) and acute myeloid leukemia (AML) that it is believed to represent the largest HSCT cohort of leukemia patients in the Middle East. In the study presented here, data were collected from 587 patients with AML (aged <60 years) who underwent bone marrow transplantation in the Hematology–Oncology and Stem Cell Transplantation Research Center, Shariati Hospital, Tehran, Iran between 1991 and 2011. All of the written informed consents (No: 124/911019) for the hematopoietic cell collection and transplantation were obtained from patients and donors. Their median follow-up time after transplantation was 517 days. All patients’ records were reviewed and any occurrence of adverse events including GVHD, AML relapse or regimen-related toxicities was recorded.

**Preparative regimen**

The preparative regimen for all patients was Busulfan (4 mg/kg/day administered orally on days-6 to-3) and Cyclophosphamide (60 mg/kg/day by intravenous infusion on days-2 to -1) with subsequent infusion of donor marrow cells on day zero. 

**Stem cell transplantation source (SCTs)**

Stem cell transplantation sources among our study samples included: peripheral blood (n= 552), bone marrow (n= 33) and cord blood (n= 2). Additional information is summarized in Table 1.

**Prophylaxis and treatment of GVHD**

For GVHD prophylaxis all patients received the conventional protocol of Cyclosporin (3 mg/kg/day intravenously from days-2) and Methotrexate (10 mg/m2 day + 1 and 6 mg/m2 on days 3, 6 and 11). When oral intake was possible, an oral formulation of Cyclosporin was substituted.

**GVHD incidence and grading**

The incidence of aGVHD was investigated and occurrences were graded I, II, III or IV according to the Seattle criteria.^10^^,^^11^ The incidence of cGVHD was investigated in all patients who survived for at least 90 days after transplantation.^11^^-^^13^

**Statistical analysis**

The time interval between HSCT and death from any cause related to AML or censoring was defined as Overall Survival (OS). Censoring was defined as being alive at the last follow-up. The Cox proportional hazards model was used for determination of the relationship between each variable and survival time. Furthermore, the suitability of the Cox proportional hazards model and the best functional form of the independent variables were determined using Cox- Snell residual and Martingale residuals, respectively.^14^ The proportional hazard model was used for the multivariate analysis of survival.^14^ However, because the Cox proportional assumptions for hazard were not met in defining the relationship between aGVHD grade and survival, we combined grades I and II into a single group for analysis. The incidence of cGVHD was investigated in all patients who survived for at least 90 days. The probability of OS was estimated using Kaplan-Meier estimator.^15^ Confidence intervals were calculated via Log transformation^16^ and P-value less than 0.05 was considered significant.

## Results

 Patient characteristics are given in Table 1. Of the 587 patients included in this study, 233 (39.7 %) patients were female with a mean age of 27.27 ± 12.45 years at transplant time, and 354 (60.3%) were male with a mean age of 28.34 ± 12.06 years at transplant time. The median survival time of patients was 517 days (Range: 7 - 5672 days). The first and third quartiles were 153 and 1314 days, respectively. The Cumulative incidence of cGVHD was 7, 16.8, 30.1 and 36.2 percent at 4, 6, 12, and 60 months after transplantation. overall survival (OS) rate based on Kaplan-Meier curve at 6 months, 12 months and 5 years was 87.4% (95%, CI: 84.66 -90.15), 82.2% (95%, CI: 78.87 - 85.54) and 71.9% (95%, CI: 67.40 - 76.41), respectively. The five-year survival rate was not significantly different between patients who developed aGVHD (69.7%; 95% CI: 63.82 - 75.58) and those that did not develop aGVHD (69.7%; 95% CI: 56.96 - 82.44). However, the five-year survival rate of patients who developed cGVHD was significantly higher (77.3%; 95% CI: 68.48 - 86.12) than patients who did not develop cGVHD (68.2%; 95% CI: 62.52 - 73.89). Furthermore, patients who experienced relapse had a significantly lower survival rate at 1 year (53.9%; 95% CI: 42.47 -65.47) and 5 years (30.8%; 95% CI: 18.26 - 43.35) after transplantation, compared to patients who had not experienced a relapse 1 year (87.2%; 95% CI: 84.06 - 90.34) and 5 years (78.9%; 95% CI: 74.40 - 83.41) after transplantation. According to disease stage, the five-year survival rate was76.9% (95% CI: 72 - 81.8) for the first complete remission (CR1), 65.5% (95%. CI: 51.98-79.10) for CR2 and 32.8% (95%, CI: 7.91-57.68) for CR3. The five-year overall survival was calculated for patients who developed aGVHD based on grade (І, ІІ, ІІІ and IV); 71.4% (95%, CI: 61.6 - 81.2) for grade І, 78.7% (95%, CI: 70.47-86.94) for grade ІІ, 57.1% (95%, CI: 43.19 - 71.02) for grade ІІІ and 40% (95%, CI: 37.28-42.73) for grade IV. Overall survival after relapse was 53.9% (95%, CI: 42.34 - 65.47) at 1 year and 30.8% (95%, CI: 18.26 -43.35) at 5 years. Cumulative hazard of relapse at 6 months, 1 and 5 years was 10.4 ± 0.014, 14.8 ± 0.016and 20.1 ± 0.021, respectively.

No significant relationship was observed between age at transplant time and survival (P= 0.74). Mean age of patients at transplant time for who survived or died was 27.83 ± 12.02 and 28.23 ± 12.3, respectively, but there was a significant relationship between hazard of death and donor sex (P=0.014), (male patients had a lower survival rate). Furthermore, a significant relationship was observed between complete remission (CR) status and survival rate. Compared to the other groups, patients categorized in CR1 had a higher survival rate (P< 0.001). 

**Figure 1:**
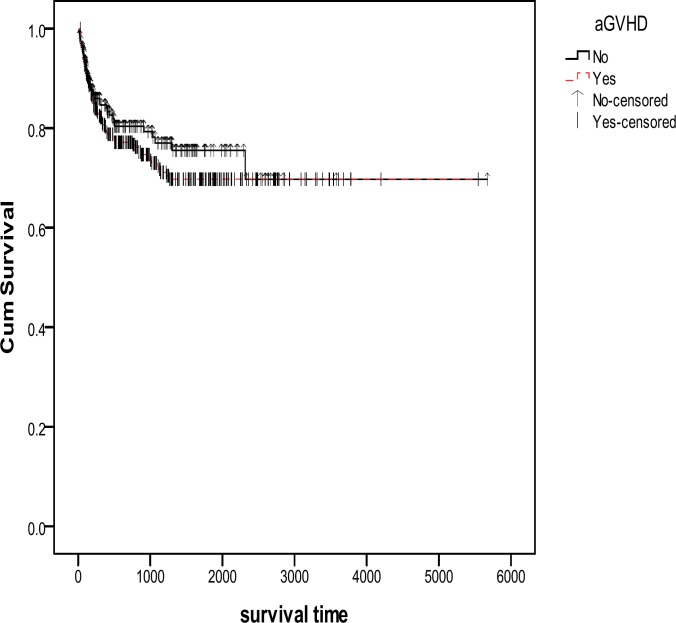
Kaplan-Meier estimated survival after transplantation for patients with AML in Iran between 1991-2011. There was no significant relationship found between the occurrence of aGVHD and survival (p=0. 33). aGVHD: acute Graft Versus Host Disease, **Yes:** Survival rate in patients who developed aGVHD, **No:** Survival rate in patients who did not develop aGVHD, **No-censored:** Time of incidence of death in patients who did not develop aGVHD, **Yes-censored:** Time of incidence of death in patients who developed aGVHD

**Figure 2:**
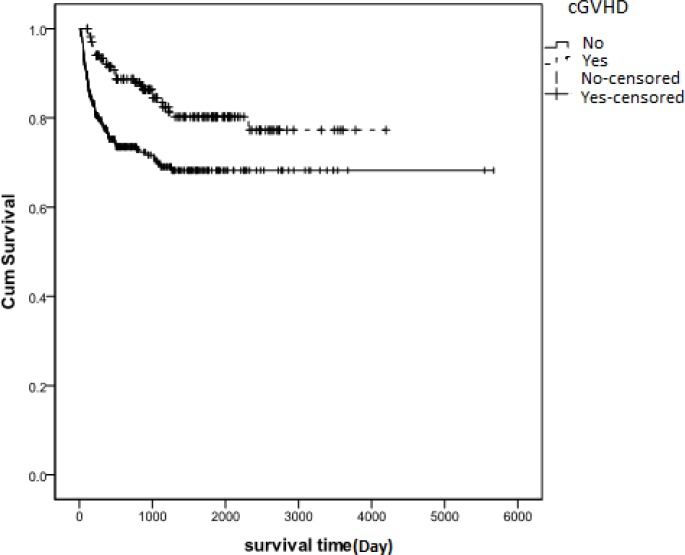
Kaplan-Meier estimated survival after transplantation for patients with AML in Iran between 1991 and 2011. Patients who developed cGVHD had a higher survival rate than patients who did not develop cGVHD (p<0.001). **cGVHD**: chronic Graft Versus Host Disease, **Yes**: Survival rate in patients who developed cGVHD, **No**: Survival rate in patients who did not develope cGVHD, **No-censored**: Time of incidence of death in patients who did not develope cGVHD, **Yes-censored**: Time of incidence of death in patients who developed cGVHD

Results showed that aGVHD developed in 318 (54.2%) patients. The incidence of cGVHD among patients who survived 90 days or longer after transplantation was 29.1% (n= 171). We found no significant relationship between survival time and aGVHD (P= 0.33) (Figure1). However, we found a significant relationship between cGVHD and overall survival (P=0.001, HR= 0.476, 95%), indicating that cGVHD had a protective effect on patient survival rate (Figure 2). Among patients who developed aGVHD, 38.7% showed cGVHD. Hazard of death in patients with both aGVHD and cGVHD was less than those who did not develop cGVHD and aGVHD (HR= .629, P= 0.078) (Table 2). Overall survival after relapse was 53.9% (95%, CI: 42.34 - 65.47) at 1 year and 30.8% (95%, CI: 18.26 - 43.35) at 5 years. Cumulative hazard of relapse at 6 months, 1 and 5 years was 10.4 ± 0.014, 14.8 ± 0.016 and 20.1 ± 0.021, respectively.

No significant relationship between aGVHD grade and survival was detected, using log-rank test (P= 0.56). Therefore, we combined grade I and II and re-analyzed data, using the Cox proportional hazard test (Table 2). 

**Table 1:** Prognostic factors of overall survival (OS) in patients with AML who received transplantation in Iran between 1991 and 2011

** Variable**		**Survive Number** **(%)**	**Dead Number** **(%)**	**Total Number ** **(%)**	**P**
**aGVHD**	Yes	237 (74.5)	81 (25.5)	318 (100.0)	0.33
	No	224 (83.3)	45 (16.7)	269 (100.0)	
**Grade aGVHD**	0^*^	225 (83.3)	45 (16.7)	270 (100.0)	0.56
	Ι	82 (75.9)	26 (24.1)	108 (100.0)	
	II	101 (82.1)	22 (17.9)	123 (100.0)	
	III	47 (65.3)	25 (34.7)	72 (100.0)	
	IV	6 (42.9)	8 (57.1)	14 (100.0)	
**cGVHD**	Yes	143 (83.6)	28 (16.4)	171 (100.0)	P<0.001
	No	318 (76.4)	98 (23.6)	416 (100.0)	
**Source of SCTs**	BM	19 (57.6)	14 (42.4)	33 (100.0)	0.004
	Cord	1 (50.0)	1 (50.0)	2 (100.0)	
	PB	441 (79.9)	111 (20.1)	552 (100.0)	
**Relapse**	Yes	44 (48.9)	46 (51.1)	90 (100.0)	P<0.001
	No	417 (83.9)	80 (16.1)	497 (100.0)	
**CR**	CR1	356 (82.03)	78 (17.97)	434 (100.0)	P<0.001
	CR2	75 (78.13)	21 (21.87)	96 (100)	
	CR3	10 (43.48)	13 (56.52)	23 (100.0)	
**Donors Type**	HLA Mismatch, sibling/other relative	6 (31.6)	13 (68.4)	19 (100.0)	P<0.001
	Other relative, HLA matched	9 (75.0)	3 (25.0)	12 (100.0)	
	Sibling, HLA matched	446 (80.4)	109 (19.6)	555 (100.0)	
	Unrelated	0 (0.0)	1 (100.0)	1 (100.0)	
**GVHD**	Did not develop aGVHD or cGVHD	181 (81.9)	40 (18.1)	221 (100.0)	P<0.001
	Developed aGVHD or cGVHD	180 (74.1)	63 (25.9)	243 (100.0)	
	Developed both aGVHD and cGVHD	100 (81.3)	23 (18.7)	123 (100.0)	

**Figure 3:**
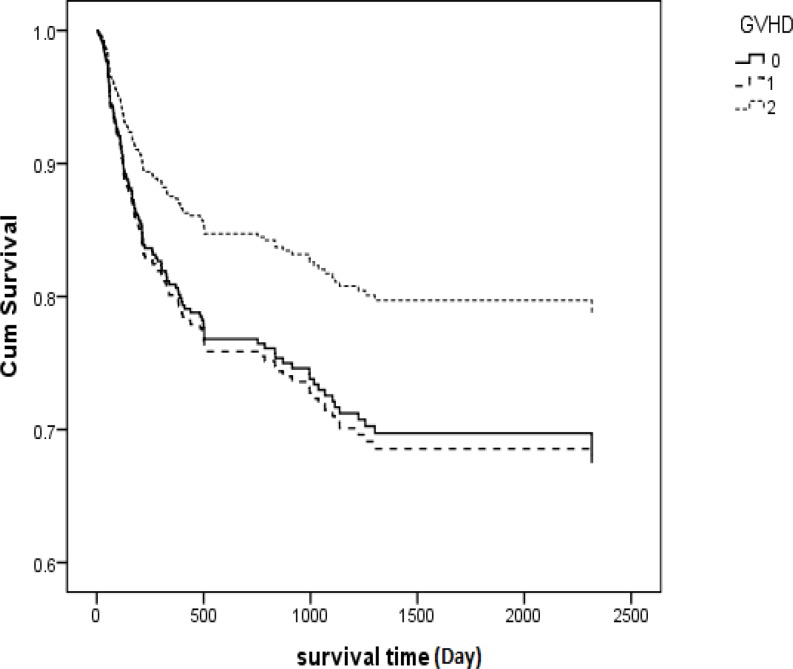
Kaplan-Meier estimated survival based on GVHD status for AML patients who received transplantation in Iran between 1991 and 2011. There was a significant relationship between survival and grade-aGVHD (p<0.001). **GVHD**: Graft Versus Host Disease, 0: did not develop aGVHD or cGVHD, **1: ** Developed aGVHD or cGVHD and **2: ** developed both aGVHD and cGVHD

A significant relationship between overall survival and grade of aGVHD was detected in patients (P<0.001), with patients experiencing grade IV aGVHD having a survival rate much worse than any other grade of aGVHD (five-year survival rate was only 42.9% in grade IV). Hazard of death in patients experiencing aGVHD grade ІІІ and IV was 1.8 and 4.2 times, respectively, more than patients in the reference group (Table 2). Hazard of death for patients who developed both aGVHD and cGVHD was 0.629 compared with the reference group (Patients who did not develop either aGVHD or cGVHD) (P= 0.78) (Table 2, Figure 3). Hazard of death for patients who developed both aGVHD and cGVHD was 0.629 compared with the reference group (patients who did not develop either aGVHD or cGVHD) (P= 0.78) (Table 2, Figure 3). 

**Figure 4:**
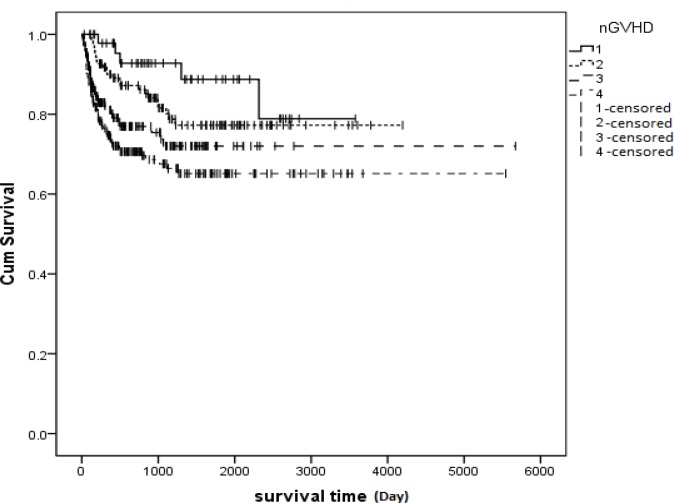
Kaplan-Meier estimated relationship between GVHD and Hazard of Death in AML patients who received transplantation in Iran between 1991 and 2011. There was a significant relationship between survival and GVHD (p=0.002). **Group 1**: The patients who developed cGVHD only, **Group 2**: The patients who developed both aGVHD and cGVHD, **Group 3**: The patients who did not develop either aGVHD or cGVHD and **Group 4**: The patients who only developed aGVHD, **1-censored**: Time of incidence of death in group 1, **2-censored**: Time of incidence of death in group 2, **3-censored**: Time of incidence of death in group 3, **4-censored**: Time of incidence of death in group 4

**Figure 5:**
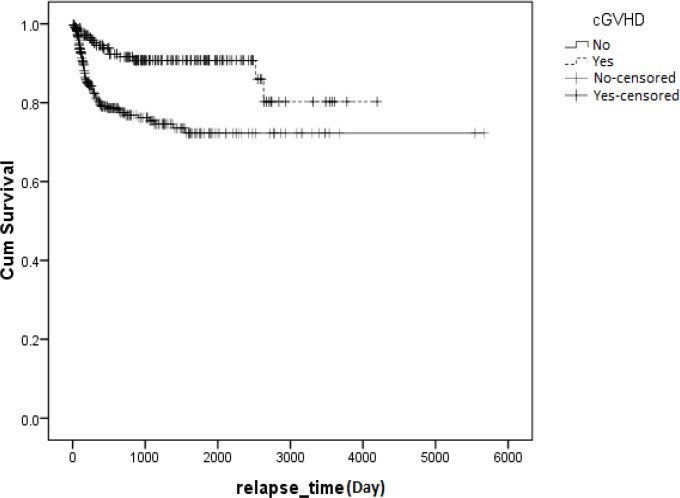
Kaplan-Meier estimated relationship between cGVHD and relapse time for AML patients who received transplantation in Iran between 1991 and 2011. There was a significant relationship between survival and relapse time (p<0.001). Chronic Graft Versus Host Disease, **No: **Survival rate for patients who did not develope cGVHD, **Yes:** Survival rate for patients who developed cGVHD, **No-censored:** Time of incidence of death in patients who did not develope cGVHD, **Yes-censored**: Time of incidence of death in patients who developed cGVHD

Another categorization for this purpose is shown in Figure 4.No significant relationship was observed between aGVHD and relapse (P= 0.285), but a significant relationship was seen between cGVHD and relapse (P<0.001). Patients who developed cGVHD showed less relapse or a better survival rate (Figure 5).

**Table 2:** Relationship between some variables with survival by using univariateand and multivariate Cox regression in patients with AML in Iran (1991-2011)

** Variable**				** HR**	**%** ** 95 CI for HR**		** P**
					** Lower**	**Upper**	
**Univariate Cox Regression**	a **GVHD Yes/No**			1.198	0.333	1.727	0.333
	a**GVHD grade**		grad І and ІІ	Ref.			
			grad ІІІ	0.007	1.182	0.007	0.007
			grad IV	P<0.000	2.050	P<0.001	P<0.001
	c **GVHD Yes/No**			0.476	0.001	0.727	0.001
	**Relapse Yes/No**			4.191	P<0.000	6.054	P<0.001
	** GVHD**	Did not develop aGVHD or cGVHD		Ref.			
		Developed aGVHD or cGVHD		0.822	0.703	1.559	0.842
		Developed both aGVHD and cGVHD		0.078	0.375	1.054	0.078
**Multivariate Cox Regression**	**Relapse** c **GVHD**			3.869 0.554	P<0.000 0 .007	5.608 0.848	P<0.001 0.007

*Reference Group.

a GVHD: acute Graft versus Host Disease,

c GVHD: chronic Graft Versus Host Disease

Furthermore, no significant relationship was observed between aGVHD and survival rate (P=0.33). Meanwhile, there was no significant relationship between aGVHD grade and relapse (P=0.59).The effects of relapse and cGVHD on survival time were analyzed using the multivariate Cox regression model. This model showed that cGVHD and relapse were two independent factors for prediction of survival or death after bone marrow transplantation. H (t) = h0 (t) e1.35relape-0.59cGVHD. Hazard of death for cGVHD was estimated to be 0.554. This rate indicates the power of the cGVHD variable in increasing survival time. The estimated hazard of death was 3.869 for relapse, indicating the adverse effect of this variable on survival time (Table 2). There was a significant relationship between relapse and cGVHD. The risk of relapse for patients who developed cGVHD was 2.92 times lower than those who did not develop cGVHD (P<0.001, 95%, CI: 1.69-5). Additional information on relationship between relapse and GVHD is summarized in Table 3.

## Discussion

 HSCT has significant therapeutic benefits to patients suffering from hematologic disorders such as AML, but the benefits of the stem cell graft can be limited by the significant morbidity and mortality that can be associated with developing GVHD.^17^ Our objectives were to investigate the incidence of GVHD (acute and chronic) and any effects that GVHD might have on survival rate on a large cohort of patients with AML who underwent HSCT in Iran between 1991 and 2011. Previously we found that the incidence of cGVHD (among a smaller cohort of patients who survived for 90 days or longer after transplantation with matched sibling donors) was 25.5%.^18^^,^^19^ In the current study (a cohort of 587 patients with more heterogeneous donor type), we estimated the incidence of cGVHD to be slightly higher (29.1%).

**Table 3:** Relationship between aGVHD and cGVHD and survival according to relapse status in patients with AML who received transplantation in Iran between 1991 and 2011

**Relapse**	** Variable**		**Survival Number (%)**	**Deceased Number (%)**	**Total Number (%)**	**P**
**Yes**	a **GVHD**	Yes	21 (42.0)	29 (58.0)	50 (100.0)	0.43
		No	23 (57.5)	17 (42.5)	40 (100.0)	
	c **GVHD**	Yes	8 (50.0)	8 (50.0)	16 (100.0)	0.29
		No	36 (48.6)	38 (51.4)	74 (100.0)	
**No**	a **GVHD**	Yes	216 (80.6)	52 (19.4)	268 (100.0)	0.34
		No	201 (87.8)	28 (12.2)	229 (100.0)	
	c **GVHD**	Yes	135 (87.1)	20 (12.9)	155 (100.0)	0.01
		No	282 (82.5)	60 (17.5)	342 (100.0)	

a **GVHD**: acute Graft versus Host Disease,

c **GVHD**: chronic Graft Versus Host Disease

However, we did not observe any significant relationship between survival time and aGVHD (P= 0.33) in the present study, which is consistent with the results of the previous smaller study in which we found that the occurrence of aGVHD resulted in a negative (but not significant) effect on overall survival (P= 0.11, HR = 0.59).^18^

Although cGVHD is usually considered to harbor the beneficial graft-versus-leukemia effect, it still appears to remain as a single major determinant of long-term outcome and quality of life following allogeneic transplantation.^20^ Based on our study results, a significant relationship exists between survival and cGVHD; however, the literature is somewhat conflicting. In a study that was conducted by Kataoka et al. no improvement in survival rate was observed in patients who developed cGVHD,^21^ however, in our previous studies we observed a significant relationship between cGVHD and overall survival (P<0.001 exp (b) = 3.66)^18^. In that study, the results indicated that OS was about 3.11 times longer in the AML patients who developed cGVHD compared to the patients who did not develop cGVHD^18^.cGVHD is largely predicted by the prior occurrence of aGVHD.^22^

developed cGVHD,^21^ however, in our previous studies we observed a significant relationship between cGVHD and overall survival (P<0.001 exp (b) = 3.66).^18^ In that study, the results indicated that OS was approximately 3.11 times longer in the AML patients who developed cGVHD compared to the patients who did not develop cGVHD.^18^ cGVHD is largely predicted by the prior occurrence of aGVHD.^22^ Chronic GVHD is an important clinical problem after bone marrow transplantation, and it is important to understand if there is any relationship between aGVHD and cGVHD in terms of both prevention and management. We, therefore, analyzed the relationship between cGVHD and aGVHD in our cohort. Among patients who developed aGVHD, 38.7% showed cGVHD Hazard of death in these patients was less than those who did not experience cGVHD and aGVHD (HR=0.629, P=0.078). However, the relationship between these two variables is complex - some data suggest that cGVHD is an extension of aGVHD, while others suggest it is a distinct entity.^23^

Acute and chronic GVHD have been presented as favorable in decreasing the risk of AML relapse in some studies,^24^ although in others, results indicated that the relapse rate decreased when only aGVHD was present.^25^ In our current study we did not find any significant relationship between aGVHD and relapse (P=0.285). Although cGVHD causes adverse effects, it has also been associated with decreased risk of leukemia relapse in a study by Lee et al.^26^ Our study supports these findings with a significant relationship observed between cGVHD and relapse (P<0.001). In the current study, patients who developed cGVHD had better survival rates. In patients who developed cGVHD, the risk of relapse was 2.92 times lower than patients who did not develop cGVHD (P<0.001, 95%, CI: 1.69-5). Several observational studies have also demonstrated that cGVHD is associated with lower relapse rates.^26^^-^^30^ In yet another study, cGVHD was associated with a lower relapse risk in all diagnoses.^31^ Kim et al. (2007) did not find any association between relapse rate with cGVHD.^25^

In a smaller AML cohort using a multivariate model, overall survival had a strong relationship with relapse (exp (b) = 10.58, P<0.001).^18^ Our current study indicates that a hazard of death score in patients who relapsed was 3.869 times worse than patients who had not relapsed, supporting the relationship found in the smaller study. The overall survival (OS) rate based on Kaplan-Meier curve in our AML patient cohort with the median survival time of 517 (17.2 months) days in 6 months, 1 and 5 years was 87.4% (95%, CI: 84.66 - 90.15), 82.2% (95%, CI: 78.87 - 85.54) and 71.9% (95%, CI: 67.40 -76.41), respectively. The 5-year survival reported in our study is much longer than that reported in other studies e.g. the 3-year survival calculated by Baron et al. was 54 ± 1%,^32^ and the 5-year overall survival reported by Mitus et al. for their entire cohort of patients was 55%^33^ from date of diagnosis. The 5-year survival rate reported in our study is similar to those previously reported by us based on smaller sample size, it was 65% (95% CI: 60.7 - 69.3).^18^^,^^19^ Additionally, we found that the patients in our current cohort had higher median survival time compared to other studies (17.2 Vs. 9 months).^34^

Based on our study, a significant relationship between overall survival and aGVHD grade was detected (P<0.001), so those patients who experienced grade IV aGVHD had a much lower survival rate than patients who experienced milder grades of aGVHD. In another study, significant improvement was detected in overall survival among AML patients who developed grade I acute GVHD (P = 0.0002).^6^ Relapse rate was lower in grade I acute GVHD than in grade II.^6^ Moreover, in a similar study performed by Baron et al, it was shown that grade I aGVHD was associated with a lower risk of relapse (hazards ratio (HR) = 0.7, P= 0.02. grade II aGVHD had no net impact on OS, while grade III-IV aGVHD was associated with a worse OS (HR= 0.4, P<0.0.001) owing to high risk of non-relapse mortality (NRM; HR= 5.2, P<0.0001).^32^ However, in our study, we were unable to find any significant relationship between these variables (P=0.39). Five-year overall survival in patients with aGVHD (I, II, III and IV) was 71.4% (95%, CI: 61.6 -81.2), 78.7% (95%, CI: 70.47 - 86.94), 57.1% (95%, CI: 43.19 - 71.02) and 40% (95%, CI: 37.28 - 42.73), respectively, while in the similar study, 4-year OS was 66 ± 2% in patients with grade I acute GVHD, 60 ± 2% in patients without acute GVHD, 56 ± 4% in patients with grade II acute GVHD and 43 ± 4% in patients with grade III–IV acute GVHD.^32^

No significant relationship was seen between age at transplant time and survival (P= 0.74), which is consistent with previous studies.^19^ Based on the results of our study, there was a significant relationship between donor sex and hazard of death (P=0.014). This is also consistent with our previous findings.^19^

Overall Survival (OS) after relapse was 53.9% (95%, CI: 42.34 - 65.47) and 30.8% (95%, CI: 18.26 - 43.35) at 1 and 5 years, respectively, much higher than what was reported in another study in which the OS after relapse was as low as 19.3% at 3 years.^35^

Our results suggest that cGVHD has a positive influence on the survival rate of patients with AML. However, based on our findings, prior occurrence of aGVHD in patients who later developed cGVHD could result in an increase in the hazard of death score. We recommend that further work needs to be done to investigate this link and whether it might influence patient survival. 
